# How narrative transportation in movies affects audiences’ positive word-of-mouth: The mediating role of emotion

**DOI:** 10.1371/journal.pone.0259420

**Published:** 2021-11-05

**Authors:** Shih-Tse Wang, Yao-Chien Tang

**Affiliations:** Graduate Institute of Bio-Industry Management, National Chung Hsing University, Taichung, Taiwan, Republic of China; University of Connecticut, UNITED STATES

## Abstract

On the basis of the cognitive–affective–behavioral model, this study investigated the effects of narrative transportation in movies on audience emotion and positive word-of-mouth (PWOM). To the best of the authors’ knowledge, this is the first study to explore the effects of the multidimensionality of narrative transportation on the multidimensionality of emotion. In this study, narrative transportation was divided into dimensions of empathy, immersion, and belief revision, and emotion was divided into pleasure and arousal. To explore the relationship between narrative transportation, emotions, and PWOM, the participants, comprising individuals with movie watching experience, completed a questionnaire on the movie that had left the deepest impression on them. The responses were analyzed through structural equation modeling. Empathy, immersion, and belief revision were significantly and positively associated with pleasure and arousal, which positively correlated with PWOM. The relationship between empathy or immersion and PWOM was partially mediated by pleasure and arousal, whereas pleasure and arousal fully mediated the association between belief revision and PWOM. Practical suggestions for filmmakers were derived from the present findings.

## Introduction

In 2019, global box office revenues for movies grossed US$42.2 billion, up 10% from 5 years ago [1]). Yang and Yao [2] indicated the growing importance of movies to the entertainment industry. The movie industry is risky [3] because both profits and losses are high [4]. Amid the boom observed over the past few years, consumer behaviors in movies, such as product placement, have received considerable scholarly attention [5–8]. Over the past decade, numerous studies have explored contributing factors to movie box office revenue, including star power [9–14], sequels [13, 14], awards [11, 15, 16], genre [17], type of movie release [18], novel adaptations [19], and criticism [12, 13, 15, 16].

Word of mouth (WOM) is frequently considered the most critical determinant of movie success [20, 21]. Researchers have suggested that positive word-of-mouth (PWOM) among audiences positively influences movies’ gross box office revenue [22] and that positive emotions are the optimal predictor of PWOM [23]. Thus, filmmakers must deepen their understanding of what facilitates positive emotions and PWOM among audiences. Narrative transportation, which refers to cognitive and emotional engagement in a story [24], is positively associated with consumers’ positive emotions [25] and likelihood of engaging in WOM [26]. Thus, this study investigated the facilitators of audiences’ positive emotions and PWOM from the perspective of narrative transportation. Specifically, on the basis of the widely used cognitive–affective–behavioral model of attitude [27], narrative transportation can be reasonably supposed to affect audiences’ emotional responses to a movie, which in turn shapes their intentions to engage in PWOM. This study presents a conceptual research framework for understanding the PWOM intentions of movie audiences based on this model, where cognitive, affective, and behavioral intention refer to beliefs regarding a movie’s narrative transportation, emotions toward the movie, and PWOM intentions, respectively.

A study suggested that narrative transportation contains multiple dimensions [28]. Because empathy is one of the main components of narrative transportation [29], narrative transportation is defined as immersion in a story, [25] and the term ‘belief revision’ describes how the story can modify people’s beliefs [30], we used these three dimensions to assess narrative transportation: empathy, immersion, and belief revision. Moreover, pleasure and arousal were considered dimensions of emotion. The present study investigated the effects of the multidimensionality of narrative transportation on that of emotion, which to the best of the authors’ knowledge have not yet been explored. The effects of the multidimensionality of emotion on PWOM were further examined. Specifically, a mediation model explaining the effects of narrative transportation on PWOM through emotion was tested. The impacts of various facets of narrative transportation (i.e., empathy, immersion, and belief revision) on various types of emotions (i.e., pleasure and arousal) and PWOM were examined. Understanding of these connections can provide practical guidance for increasing PWOM behavioral intention through plot development.

## Literature review and hypotheses

Narrative transportation is a mixed experience involving attention and imagery [31]. Specifically, it occurs when a person is immersed in the illusionary world evoked by a narrative because of empathy for the characters [32]. Narrative transportation can diminish negative cognitive responses and emotional reactions to achieve persuasive functions [25]. Studies have evaluated the effects of narrative transportation in advertisements on purchase intention and brand attitude [33–35]. Narrative transportation can also be considered a state of mental simulation [36]. For instance, novel readers and theater and movie audiences alike experience real sorrow upon the death of a favorite character (e.g., a superhero), despite the fact that it is fictional [32]. The concept of narrative transportation, which centers on the experience of stories, is multifaceted [28]. In this study, narrative transportation was divided into dimensions of empathy, immersion, and belief revision. Empathy describes one’s ability to understand and share others’ feelings, thoughts, and experiences [37]. Immersion refers to one’s degree of involvement in an activity [38]. Belief revision is defined as changes in one’s beliefs in certain circumstances [39].

Mehrabian and Russell [40] proposed that emotional states are governed by three basic domains, namely pleasure, arousal, and dominance, variations in which correspond to emotional responses [41]. This model can explain how emotion is physically experienced and expressed [42] and the effects of stimuli on individual intentions and behaviors [43]. However, Mohammed and Althonayan [44] suggested that dominance should be removed from this model. As Russell and Pratt [45] demonstrated, dominance constitutes only a small proportion of emotion, and pleasure and arousal sufficiently represent emotional responses. Pleasure, the extent to which an individual feels good, joyful, or happy in specific situations [44], contains both physical and psychological elements [46]. Arousal is defined as the degree to which one feels stimulated, alert, active, or excited [47].

### Effects of empathy on pleasure and arousal

According to Toremen et al. [37], empathy refers to the ability to perceive matters from others’ points of view and understand their feelings. Pleasure indicates the degree to which a person feels joyful, happy, or satisfied in a situation [48]. A Study has suggested a correlation between empathy and rated experiences of pleasure [49]. Notably, empathy is an approach-related affective state regardless of whether the stimulus is negative or positive. Low-empathy individuals focus on the negative aspects of an experience, whereas high-empathy individuals tend to focus on the positive aspects [50]. Audiences experience pleasure when they feel that they are becoming closer to certain characters [51]. Empathy involves understanding others’ thoughts [52]. Arousal refers to an individual’s level of excitement or stimulation [47]. Researchers have suggested that higher levels of empathy are linked with changes in physiological arousal [53] and that the expression of cognitive empathy triggers immediate emotional arousal [54]. Conversely, low physiological arousal could be indicative of a lack of empathy [53]. High empathy may correspond with high emotional arousal. One study reported that service empathy was positively correlated with pleasure and arousal in customers [44]. Thus, the following hypotheses are proposed:

Hypothesis 1: Empathy is positively related to pleasure.

Hypothesis 2: Empathy is positively related to arousal.

### Effects of immersion on pleasure and arousal

Immersion is the degree of one’s involvement in a situation [38] and is linked to the pursuit of joy and escape from real-life stresses [55]. In the present study, pleasure is defined as one’s level of joy or happiness when expectations are met. A study noted that audiences experience pleasure when they feel immersed in a show’s world [51]. Hence, people may feel happy when they are immersed in a movie. Immersion refers to one’s mental focus on a story and eagerness or impatience to know its ending. Arousal is a subjective state of excitement or stimulation [56]. Immersion may directly affect audiences’ emotions with regard to arousal [57]. Immersion in a movie may trigger emotional arousal. Thus, this study advances the following hypotheses:

Hypothesis 3: Immersion is positively related to pleasure.

Hypothesis 4: Immersion is positively related to arousal.

### Effects of the belief revision on pleasure and arousal

Belief revision is the changing of beliefs when one is presented with new information [39].

Many people are interested in staying abreast of the latest trends in fashion, style, or innovation. One reason that people enjoy consumption is that they develop new ideas and learn about trends and movements when they see new items [58]. One study asserted that shoppers seek fun and entertainment through the acquisition of new information from the consumption environment [59]. People are willing to collect information about new fashions and trends; moreover, they are interested in trying new things, generating new ideas, and experiencing excitement, stimulation, and adventure [60, 61]. Thus, belief revision during movie viewing may correspond to a heightened state of pleasure and arousal. This study proposes the following hypothesis:

H5: Belief revision is positively related to pleasure.

H6: Belief revision is positively related to arousal.

### Effects of pleasure on PWOM

WOM, defined as communication about a brand, product, organization, or service between a perceived noncommercial sender and a receiver [62], contributes critically to what people know, feel, and do [63]. It can reflect the sender–receiver relationship and change the attitude and behavior of the receiver through interpersonal communication [64]. Pleasure, which refers to the intensity of happiness or enjoyment in certain situations [44], is experienced when one loves something. Sharing the source of one’s pleasure with others positively affects PWOM [43]. More specifically, when a movie gives someone pleasure, they may be willing to recommend it to others. Thus, the following hypothesis is proposed:

Hypothesis 7: Pleasure is positively related to PWOM.

### Effects of arousal on PWOM

As defined by Shin et al. [47], arousal is the degree to which a person feels stimulated or excited. WOM is a type of person–person communication [64]. In the present study, PWOM refers to people’s tendencies to recommend or speak positively of something they enjoy. Talking with others helps people return from an excited to a neutral state [65]. As Huang et al. [43] indicated, arousal positively affects WOM. Similarly, Feng [66] reported that high-level arousal led to higher WOM than low-level arousal. If a movie generates intense excitement in a viewer, they may be willing to recommend it to others. Thus, the following hypothesis is proposed:

Hypothesis 8: Arousal is positively related to PWOM.

### Mediating role of emotions in the effects of narrative transportation on PWOM

With narrative transportation as an outcome of exposure [67], the cognitive–affective–behavioral model of attitude highlights how features of external stimuli can lead to psychological consequences [68]. Mental simulation, which commonly occurs in response to stories or narratives, is defined as the cognitive construction of hypothetical scenarios [25]. Furthermore, narrative transportation is a facilitator of emotional effects [67]; the more narrative transportation that is evoked, the more positive emotions consumers generate [69]. Researchers have indicated that narrative transportation triggers a sense of pleasure and fun [34, 70]. Narrative transportation also promotes experiential processing and heightens emotional arousal, which serve as an impetus for action-taking [71]. One study observed that external stimuli can generate pleasure and arousal, which in turn triggers approach–avoidance behaviors [72]. Another study reported that arousal and pleasure mediated the effects of external stimulus–related cognitions on outcomes in customers [73]. Thus, we propose the following hypothesis.

Hypothesis 9: Emotions mediate the association between narrative transportation and PWOM.

**Fig 1:**
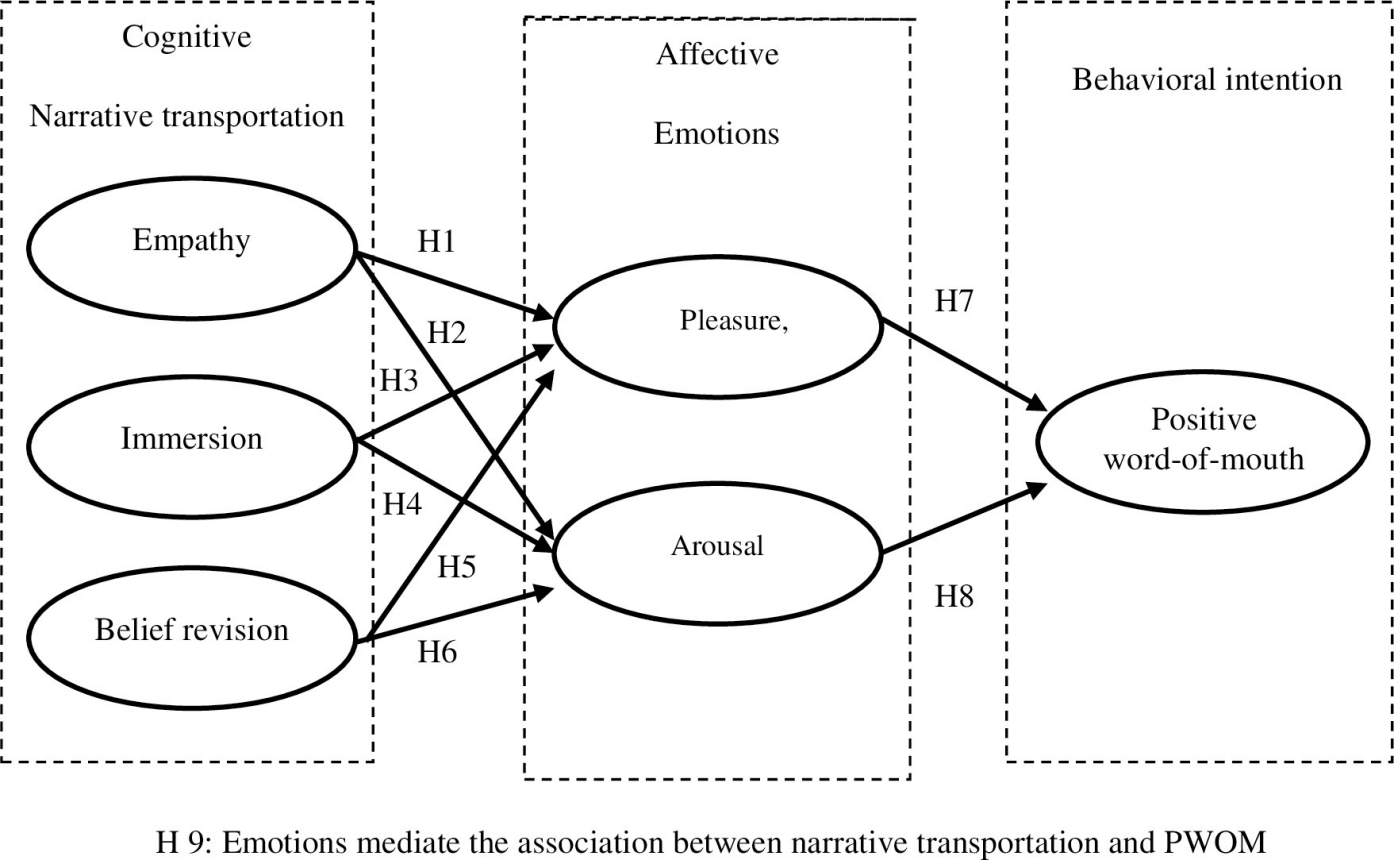
Conceptual research framework.

Fig 1 presents the research framework.

## Research methodology

### Ethics statement

The participants comprised individuals who were invited to complete the survey. Participants were informed of the study objectives and of the confidentiality and anonymity of their responses. Prior to study commencement, informed consent was obtained from all the participants online. The questionnaire content had no sensitive information. The names of the participants were not collected. The responses were anonymized to prevent the results from being linked to any individual. No ethical concerns aside from preserving the participants’ anonymity were involved, as is standard in socioeconomic studies.

### Sample and data collection

Cinema in Taiwan is in its golden age. Many people watch movies as a leisure activity [74]. The participants of the present study were people who had watched a movie in any form. Before the formal questionnaire was distributed, a pretest questionnaire was given and modifications to potentially unclear language were made according to their suggestions. Convenience sampling was used for participant selection; the questionnaire was accessible between October 2 and October 22, 2019, through a link posted on social media platforms Facebook and Line. The 404 respondents (women 57.9%, men 42.1%) were asked about the movie that had left the deepest impression on them. All 404 questionnaires collected were valid. Regarding the age distribution, individuals aged 20–29 years accounted for the largest proportion (44.3%), followed by those aged 30–39 years (26.7%). These results are roughly consistent with those from a study by the Motion Picture Association of America [1] that reported that individuals aged between 25 and 39 years viewed movies the most frequently. Regarding education level, individuals with college or university degrees constituted the largest proportion (60.1%), followed by those with a master’s degree or higher (28.0%).

### Scale development

The 15 questionnaire items were formulated from the extant literature. Seven items on narrative transportation were adapted from Knowles and Linn [52] and divided into one 3-item and two 2-item scales. Three items on pleasure were adapted from a 2014 study by Petzer et al. [75]. The 2-item scale on arousal developed by Hsieh [56] was modified. PWOM was assessed using three items taken directly from Ulker-Demirel et al. [20]. The questionnaire was scored on a 7-point Likert scale, and the anchors ranged from 1 (strongly disagree) to 7 (strongly agree). The questionnaire items are presented in Table 1.

**Table 1:** Results of the internal reliability and convergent validity tests.

Latent variable	Observed	Factor loading	Mean	SD	CR	AVE
	Variables					
Empathy	I find myself thinking about what the characters may be thinking.	0.85	5.46	1.34	0.90	0.76
	I find that I can easily take the perspective of the character(s).	0.87	5.21	1.36		
	I find myself feeling what the characters may be feeling.	0.89	5.30	1.33		
Immersion	I get mentally engaged in the movie.	0.91	5.85	1.14	0.89	0.81
	My mind is often focused on the movie.	0.89	5.83	1.20		
Belief revision	I often found that the movie has an impact on how I see things.	0.90	4.83	1.63	0.83	0.71
	I found myself accepting storylines that I might have otherwise considered to be unrealistic.	0.78	4.40	1.64		
Pleasure	I love this movie.	0.85	5.86	1.17	0.90	0.75
	Watching this movie gave me great pleasure.	0.95	5.91	1.16		
	This movie met my expectations.	0.80	5.72	1.22		
Arousal	I felt stimulated when I was watching this movie.	0.91	5.28	1.42	0.79	0.66
	I felt excited when I was watching this movie.	0.70	4.94	1.45		
Positive word-of- mouth	I say positive things to other people about this movie.	0.85	5.57	1.33	0.93	0.82
	I would recommend this movie to anyone who asks my opinion.	0.93	5.78	1.24		
	I would recommend this movie to my friends.	0.93	5.75	1.26		

SD, standard deviation; CR, composite reliability; AVE, average variance extracted.

## Results

LISREL 8.7 software and structural equation modeling (SEM) were used to examine the model fit and the causal relationships between the variables. SEM, which combines path and factor analyses, comprises two components: the measurement model and the structural model. Also known as confirmatory factor analysis, the measurement model investigates the relationships between observed and potential variables and is used to verify whether the measurement model shows a good reliability and validity [76]. Relationships between potential variables can be tested using the structural model. Part of construct validity, convergent validity measures the level of consistency between latent variables [77]. It is determined using item factor loadings, composite reliability (CR), and average variance extracted (AVE). According to Hair et al. [78], loadings greater than 0.4 can be considered for analysis, and items are considered satisfactory when their loadings exceed 0.7. Fomell and Larcker [79] proposed 0.7 and 0.5 as minimum threshold values for CR and AVE, respectively. The observed values of the factor loadings, CR, and AVE all met these requirements, ranging between 0.78 and 0.94, 0.83 and 0.93, and 0.64 and 0.82, respectively. These results indicate satisfactory internal reliability and convergent validity. Discriminant validity, a subtype of construct validity, measures the extent of differences between latent variables and other constructs. It is satisfactory when squared AVE values exceed the correlation coefficients between constructs [78]. As shown in (the gray-highlighted numbers in) Table 2, this condition was fulfilled, indicating adequate discriminant validity.

**Table 2:** Correlations among the latent variables.

Latent Variable	Correlation							
	Mean	SD	EM	IM	BR	PL	AR	PWOM
Empathy (EM)	5.32	1.21	0.87					
Immersion (IM)	5.84	1.12	0.55**	0.90				
Belief revision (BR)	4.62	1.50	0.49**	0.43**	0.84			
Pleasure (PL)	5.83	1.07	0.54**	0.63**	0.43**	0.86		
Arousal (AR)	5.11	1.29	0.48**	0.44**	0.48**	0.54**	0.81	
Positive word-of-mouth (PWOM)	5.70	1.20	0.58**	0.59**	0.42**	0.78**	0.50**	0.91

Note.

The gray-highlighted values are squared average variance extracted of each construct; the remaining values are correlation coefficients between constructs.

** *p* < 0.01.

### Structural model test

Structural models measure relationships among latent variables. The chi-square (χ^2^)/degrees of freedom (d.f.) ratio, goodness-of-fit index (GFI), adjusted GFI (AGFI), and root mean square error of approximation (RMSEA) should be below 5 [80], above 0.8 [81], above 0.8 [82], and below 0.08 [83], respectively. In the present study, these absolute fit measures were as follows: χ^2^ = 237.62 d.f. = 79, χ^2^/d.f. = 3.01, GFI = 0.93, AGFI = 0.89, and RMSEA = 0.071. The incremental fit measures, which must all exceed 0.9 [84], were as follows: comparative fit index = 0.99, normed fit index = 0.98, non-normed fit index = 0.98. The parsimonious normed fit index and the parsimonious goodness-of-fit-index, which must exceed 0.5 [85], were 0.74 and 0.61. Overall, the structural demonstrated acceptable fit.

### Hypothesis testing

The results indicate that empathy is positively correlated with pleasure and arousal, confirming hypotheses 1 and 2. Immersion is positively related to pleasure and arousal, confirming hypotheses 3 and 4. Belief revision is significantly and positively associated with pleasure and arousal, confirming hypotheses 5 and 6. Pleasure and arousal are significantly and positively associated with PWOM, confirming hypotheses 7 and 8. Table 3 presents the hypothesis testing results (H1-H8).

**Table 3:** Results of hypothesis testing (H1-H8).

Hypotheses		Standardized Path coefficients	T-value	Finding
H1: Empathy →Pleasure		0.25	3.93***	Supported
H2: Empathy →Arousal		0.24	3.49***	Supported
H3: Immersion →Pleasure		0.46	7.77***	Supported
H4: Immersion →Arousal		0.25	4.09***	Supported
H5: Belief revision →Pleasure		0.15	2.66**	Supported
H6: Belief revision →Arousal		0.37	5.78***	Supported
H7: Pleasure →Positive word-of-mouth		0.76	14.23***	Supported
H8: Arousal →Positive word-of-mouth		0.11	2.57**	Supported
	$R_{PL}^{2}=0.57$			
	$R_{AR}^{2}=0.54$			
	$R_{WOM}^{2}=0.68$			

** *p* < 0.01

*** *p* < 0.001.

The present study proposed that emotion, namely pleasure and arousal, mediates the narrative transportation–PWOM relationship. To test hypothesis 9, Baron and Kenny’s method for mediation [86], which determines whether the mediation effects between variables are partial or full, was performed. As presented in Table 4, pleasure and arousal partially mediated the effects of empathy and immersion on PWOM, whereas pleasure and arousal fully mediated the effects of belief revision on PWOM. Thus, hypothesis 9 was supported.

**Table 4:** Results of the Baron and Kenny mediation analysis.

Path	PL as a mediator				AR as a mediator			
	Step1	Step2	Step3	Step4	Step1	Step2	Step3	Step4
EM→PWOM	0.33***			0.19***	0.33***			0.28***
IM→PWOM	0.37***			0.10**	0.37***			0.33***
BR →PWOM	0.10*			0.03	0.10*			0.05
PL→PWOM			0.78***	0.60***				
EM→PL		0.23***						
IM→PL		0.45***						
BR→PL		0.12**						
EM→AR						0.24***		
IM→AR						0.19***		
BR→AR						0.28***		
AR→PWOM							0.50***	0.20***

Step 1 = IV→DV; Step 2 = IV→M; Step 3 = M→DV; Step 4 = (IV + M)→DV.

IV = independent variable; DV = dependent variable; M = mediator.

EM = empathy; IM = immersion; BR = belief revision; PL = pleasure; AR = arousal; PWOM = positive word-of-mouth.

* *p* < 0.05

** *p* < 0.01

*** *p* < 0.001.

## Discussion and conclusion

The results demonstrate that movie audiences empathize with movie characters and that immersion and belief revision are involved in the movie watching experience, which also triggers arousal. In this context, empathy, immersion, and belief revision are positively related pleasure. Furthermore, when audiences feel more pleasure and arousal during a movie watching experience, they are more likely to recommend the movie to others. The mediation analysis revealed that pleasure and arousal mediate the association between narrative transportation (i.e., empathy, immersion, and belief revision) and PWOM.

### Theoretical implications

The finding that pleasure and arousal were positively related to PWOM is consistent with that of Huang et al. [43]. Despite the assertion by Huang et al. [43] that a movie can evoke audience emotion, the influencing mechanism of narrative transportation on movie audiences’ PWOM remains unclear. Hence, the present study clarified the connection between the multidimensionality of narrative transportation on PWOM through emotion. Empathy, immersion, and belief revision were positively related to pleasure and arousal. According to the cognitive–affective–behavioral model proposed by Ajzen and Fishbein [87], the findings indicate that empathy, immersion, and belief revision, as narrative transportation dimensions, were significantly and positively associated with emotional responses in the form of pleasure and arousal, which correlated with PWOM. In addition, the mediation test revealed that pleasure and arousal mediate the association between narrative transportation and PWOM. This study contributes to the literature by aligning the evidence on narrative transportation, emotions, and PWOM.

### Practical contributions

PWOM is generally considered to be the most essential determinant of movie success [20]. The positive effects of pleasure and arousal on PWOM that were observed indicate that filmmakers should aim to produce works that evoke pleasure, stimulation, and excitement. Audiences experience pleasure when they are engaged in a movie and are eager to discover how it ends. They also experience pleasure when they can identify movie characters, think from their perspectives, and feel what they feel. Thus, filmmakers are recommended to place particular emphasis on character development [88].

As Colatrella [89] noted, information about a story, of which each individual may have different understandings, is crucial to the experience of a work. Both old and new information can provoke thought. Therefore, filmmakers should devote more attention to the messages that the story conveys. If audiences find themselves accepting storylines they initially considered to be unrealistic, pondering over other ways the story could have ended, and thinking differently after they watch a movie, that indicates that it triggered their emotional arousal. Emotional arousal is also heightened when audiences can identify the characters, think from their perspectives, and feel their feelings. In addition, screenwriters should strive to create engaging, coherent narratives, which facilitate the creation of strong connections between the audience and the story [90].

### Limitations and future directions

This study has some limitations and proposes future directions. First, the questionnaire method limited the exploration of the participants’ emotions. Future experimental studies, such as those involving electrodermal activity, an indicator of arousal [91], are therefore warranted. Second, this study investigated the effects of positive emotions (i.e., pleasure and arousal) on PWOM; we did not focus on the interaction between pleasure and arousal. One study detected an interaction between pleasure and arousal in high-pleasure environments [92]. Therefore, testing the present model with a pleasure–arousal interaction is warranted. Third, one study applied positive, negative, and neutral emotions (e.g., joy, anger, fear, sadness, and disgust) to measure consumer evaluations of emotional reactivity to commercial films. The researchers confirmed that negative emotions induced WOM [93]. Because this study focused on positive emotions, further research should be conducted including a greater number and range of emotional categories and valence—for example, bipolar dimensions from happiness to depression and from anxiety to relaxation—under the present framework. An exploration of the mediating role of emotional categories and valence would extend the understanding of the effects of narrative transportation on audiences’ emotions and the effects of those emotions on WOM. Fourth, some unmeasured variables, such as perceived similarity [94], can affect narrative transportation and should therefore be considered in future research. Fifth, the present study focused on movies; future research can explore other story-related media, such as comics, novels, or online games. Finally, because different movie genres may trigger different levels of emotional response, movies can be classified accordingly in future studies. The present findings may serve as a reference for filmmakers regarding what audiences value when they watch movies. In addition, future research on the importance of plot to the enjoyment of a movie is worthy of investigation.

## Supporting information

S1 Data (XLS)
